# Relationship of the Adherence to a Mediterranean Diet and Its Main Components with CRP Levels in the Spanish Population

**DOI:** 10.3390/nu10030379

**Published:** 2018-03-20

**Authors:** Carlos Lahoz, Elisa Castillo, Jose M. Mostaza, Olaya de Dios, Miguel A. Salinero-Fort, Teresa González-Alegre, Francisca García-Iglesias, Eva Estirado, Fernando Laguna, Vanesa Sanchez, Concesa Sabín, Silvia López, Victor Cornejo, Carmen de Burgos, Carmen Garcés, on behalf of the Investigators of the SPREDIA-2 Group

**Affiliations:** 1 Atherosclerosis Unit, Internal Medicine Department, Hospital Carlos III, Madrid 28029, Spain; josemaria.mostaza@salud.madrid.org (J.M.M.); mgalegre@uma.es (T.G.-A.); francisgiglesias@gmail.com (F.G.-I.); eva.estirado@salud.madrid.org (E.E.); flaguna.hciii@salud.madrid.org (F.L.); vanesa.sanchez@salud.madrid.org (V.S.); concesasabin@gmail.com (C.S.); silvia.lopez@salud.madrid.org (S.L.); victor.cornejo@salud.madrid.org (V.C.); 2 Lipid Laboratory, IIS-Fundación Jiménez Díaz, UAM, Madrid 28040, Spain; eli_pny@hotmail.com (E.C.); olaya.dios@quironsalud.es (O.d.D.); cgarces@fjd.es (C.G.); 3 Subdirección General de Investigación Sanitaria, Consejería de Sanidad, Madrid 28013, Spain; miguel.salinero@salud.madrid.org (M.A.S.-F.); carmen.deburgos@salud.madrid.org (C.d.B.)

**Keywords:** CRP levels, Mediterranean diet, fruits, vegetables, dairy products, fish

## Abstract

**Background:** Adherence to a Mediterranean diet seems to be inversely associated with C-reactive protein (CRP) concentration. A 14-point Mediterranean Diet Adherence Screener (MEDAS) has been developed to assess dietary compliance. **Objective:** The aim of this study was to assess whether each of the MEDAS questions as well as their final score were associated with the levels of CRP in general Spanish population. Methods: Cross-sectional analysis of 1411 subjects (mean age 61 years, 43.0% males) randomly selected from the general population. CRP levels were determined by a commercial ELISA kit. Adherence to the Mediterranean diet was measured by the 14-point MEDAS.** Results:** There was an inverse correlation between adherence to the Mediterranean diet and the CRP concentration, even after adjusting by age, gender, hypertension, metabolic syndrome, body mass index, statin treatment and hypertension treatment (*p* = 0.041). Subjects who consume ≥2 servings of vegetables per day (*p* = 0.003), ≥3 pieces of fruit per day (*p* = 0.003), ≥1 serving of butter, margarine, or cream per day (*p* = 0.041) or ≥3 servings of fish/seafood per week (*p* = 0.058) had significantly lower levels of CRP. **Conclusions**: Adherence to a Mediterranean-type diet measured by a simple questionnaire is associated with lower CRP concentration. However, this association seems to be particularly related to a higher consumption of vegetables, fruits, dairy products, and fish.

## 1. Introduction

Low-grade systemic inflammation seems to play a role in the development of obesity, metabolic syndrome, diabetes, and cardiovascular disease [1]. C-reactive protein (CRP), the best studied marker of subclinical inflammation, is an acute phase inflammatory reactant that is synthesized in the liver by the stimulation of interleukin-6 and has been proposed as a powerful predictor of cardiovascular disease. CRP concentration has been consistently associated with the risk of coronary heart disease, ischemic stroke, vascular mortality, and death [2].

Several factors influence CRP levels. Its concentration is increased in smokers and diabetics, and correlates directly with age, body mass index, triglycerides, blood pressure and inversely with exercise frequency and high-density lipoprotein (HDL)-cholesterol [3]. Diet also seems to modulate CRP levels. Consumption of healthy dietary patterns has been associated with significantly lower CRP levels [4]. In this regard, the traditional Mediterranean diet rich in olive oil, vegetables, fruits, pulses, nuts and fish, moderate in dairy products and wine, and low in red meat and processed foods has also been associated with low serum inflammatory markers, including lower CRP plasma levels [1], and low risk for cardiovascular disease [5,6].

The association between adherence to a Mediterranean diet and levels of CRP, as a marker of subclinical inflammation, has been previously investigated [7,8,9,10,11]. In all these studies, food consumption and adherence to the Mediterranean diet was determined by food frequency questionnaires (FFQ) or by self-administered 24 h recall, both time-consuming methods. The PREvencion con DIeta MEDiterranea (PREDIMED) study developed and validated a 14-point Mediterranean Diet Adherence Screener (MEDAS) to assess dietary compliance quickly and easily [12]. The possible association between CRP concentration and adherence to the Mediterranean diet using the MEDAS has only been investigated in one study including subjects with a high cardiovascular risk, giving a negative result [13].

On the other hand, there is numerous evidence reporting the association between lower CRP plasma levels and intake patterns characterized by a high consumption of vegetables and fruits in both adults and children [14,15,16]. We have also observed that fruit and vegetable consumption were the main dietary factors related to CRP levels in girls [17]. These facts suggest that maybe this particular characteristic of the Mediterranean diet could be associated with those lower CRP levels, rather than the whole dietary pattern.

Thus, the aim of this study was to assess whether each of the MEDAS questions as well as their final score were associated with the levels of CRP in a Spanish general population.

## 2. Materials and Methods

This is a substudy of a broader project, the Screening PRE-diabetes and type 2 DIAbetes (SPREDIA-2) study, which has been described in detail elsewhere [18,19]. The SPREDIA-2 study is a population-based, prospective cohort study, with baseline screening, in the Region of Madrid (Spain). A random sample of urban subjects, between 45 and 74 years, living in the northwest metropolitan area of Madrid (Spain) was selected for the study. In the reference population there are approximately 183,000 persons of this age. Pregnant women, subjects with severe chronic or terminal illnesses, institutionalized subjects or those chronically treated with steroids or antipsychotic drugs were excluded. A total of 2553 subjects were contacted for the study, of whom 1592 accepted to participate (62.4%). Of them, 1411 (88.6%) had completed the MEDAS (Mediterranean Diet Adherence Screener) and had measured CRP levels and were the ones selected for the present analysis.

Participants were scheduled in the outpatient clinic of the Hospital Carlos III after an overnight fast. Upon arrival, and after signing a consent form, a fasting blood analysis was obtained for measuring the blood levels of glucose, creatinine, and lipids and lipoproteins. Sociodemographic variables (date of birth, gender, nationality, and educational level), family history of prevalent diseases (diabetes, coronary heart disease, cerebrovascular disease), self-reported cardiovascular risk factors (smoking habit, hypertension, alcohol ingestion), comorbidities, and current treatments, were recorded in all individuals. Patients were considered current smokers if they had regularly smoked during the past 6 months.

### 2.1. Anthropometric Measurements

All participants had a physical examination with the determination of height, weight, waist circumference (midway between the lowest rib and the iliac crest), and blood pressure (the mean of the last 2 measurements after 3 determinations 5 min apart). Body mass index (BMI) was calculated as weight in kilograms divided by the square of height in meters.

### 2.2. Laboratory Methods

Glucose, creatinine, cholesterol, and triglycerides were determined by standard methods. CRP levels were determined by a commercial ELISA kit (ELISA Duoset kit, Human C-Reactive Protein/CRP, DY1707, R&D Systems, Minneapolis, MN, USA).

### 2.3. Dietary Assessment

Baseline adherence to the Mediterranean diet was measured by the 14-point Mediterranean Diet Adherence Screener (MEDAS) developed and validated by the PREDIMED study [12]. The MEDAS consists of 12 questions on food consumption frequency and 2 questions on food intake habits considered characteristic of the Spanish Mediterranean diet. Each question was scored 0 or 1. The final MEDAS score ranged from 0 to 14. The higher the score the greater the adherence to the Mediterranean diet.

### 2.4. Statistical Analysis

The normality of distribution of variables was determined with the Kolmogorov-Smirnov test. Since the CRP does not have a normal distribution, a logarithmic transformation was made for the statistical calculations, but to gain clarity, the CRP values are expressed in the text and in the tables as they were before the transformation. Quantitative variables are shown as means ± SD. Qualitative variables are shown as percentages. Comparisons between quantitative variables were performed with one-way analysis of variance (ANOVA) test, and comparisons between qualitative variables with the χ2 test. Multiple linear regression was used to evaluate the association between MEDAS score and CRP concentration after adjusting by age, gender, and those variables that in the univariate analysis showed significance levels of *p* < 0.10 (hypertension, metabolic syndrome, body mass index, statins treatment and hypertension treatment). To study the independent association of each of the questions included in the 14-points MEDAS with CRP concentration, a logistic regression analysis was used adjusting for the same variables. The statistical significance threshold was established at *p* < 0.05. Statistical processing of the data was performed with SPSS for Windows, v.19.0; IBM Corp, Armonk, NY, USA.

### 2.5. Ethical Considerations

All subjects gave written informed consent, and the study was approved by the Committee on Ethics and Research of the Hospital Carlos III (code P18/10) in Madrid. The study complied with the International Guidelines for Ethical Review of Epidemiological Studies (Geneva, 1991).

## 3. Results

Baseline characteristics of the population and by tertiles of CRP concentration are shown in Table 1. Age (mean ± SD) was 61.4 ± 6.0 years, and 43.0% were male. Diabetes was observed in 17.0% of the subjects, and 4.4% had an established cardiovascular disease (CVD). There were no differences of CRP levels by gender. Hypertension, presence of metabolic syndrome, body mass index and treatment to high blood pressure were directly associated with CRP levels, while treatment with statins was inversely associated (Table 1).

In a multivariable linear regression model, CRP concentrations were directly related with BMI (*p* < 0.001) and inversely correlated with statin treatment (*p* = 0.004) (Table 2). There was an inverse correlation between the MEDAS score (at higher score higher adherence to the Mediterranean diet) and the CRP concentration (B = −0.034, (CI95% −0.062 – (−0.005)), *p* = 0.019) even after adjusting by age, gender, hypertension, metabolic syndrome, body mass index, statins treatment and hypertension treatment (B = −0.030, (CI95% −0.058 – (−0.001)), *p* = 0.042) (Table 2).

C-reactive protein levels depending on the answer to each point of the 14-point MEDAS are shown in Table 3. Subjects who consume ≥2 servings of vegetables per day, ≥3 pieces of fruit per day, ≥1 serving of butter, margarine, or cream per day or consume ≥3 servings of fish/seafood per week had significantly lower levels of CRP. After adjusting by age, gender, hypertension, metabolic syndrome, body mass index, statins treatment and hypertension treatment, statistical significance persisted except with the consume ≥3 servings of fish/seafood per week that almost reach statistical significance (*p* = 0.058).

## 4. Discussion

In our population-based sample, including subjects between 45 and 74 years, adherence to a Mediterranean-type diet, determined by a simple questionnaire of 14 questions, was inversely related to CRP levels. These lower levels of CRP were associated with a greater consumption of some typical components of Mediterranean diet (vegetables, fruits, and fish) and dairy products.

Dietary patterns may influence the risk of diseases through the effects of CRP, a marker of subclinical inflammation and predictor of cardiovascular disease [4]. Several epidemiological studies [7,8,9,10] and clinical trials [1,11] have described an inverse relationship between adherence to Mediterranean diet and CRP concentration. Most of the studies assess adherence to the Mediterranean diet through the FFQs. These consist of a limited checklist list of foods and beverages (between 80 and 140 items) with response categories to indicate usual frequency of consumption over the period queried. FFQs are usually self-administered and require about 60 min. To reduce the waste of time in clinical practice associated with the FFQs, a 14 questions MEDAS has been developed and validated by PREDIMED study [12]. The MEDAS is a fast and simple tool to determine the adherence to the Mediterranean diet. In our population, we observed an inverse correlation between the MEDAS score and CRP concentrations after adjusting by cardiovascular risk factor and drugs. The moderate strength of this association is clinically relevant since it has been obtained only with the sum of points of a brief questionnaire and after adjusting for the factors that influence the CRP concentration. To our knowledge, only a previous study has evaluated the association between CRP concentration and adherence to the Mediterranean diet using the MEDAS. In this study, Salas et al, analyzing the first 772 participants of the PREDIMED trial, failed to find any association between adherence to the Mediterranean-type diet and concentration of CRP [13]. They also found no association between CRP concentration and a higher consumption of cereals, fruits, vegetables, fish, olive oil or alcohol. Only those participants with a higher intake of dairy products had significantly lower levels of CRP [13]. The differences between these findings and our results may be partly due to the different type of population. Their participants were subjects at high cardiovascular risk and seven years older, including a higher percentage of diabetics (64% versus 17%) and overweight and obese subjects (88.3% versus 67.8%), therefore having a lower sensitivity to detect small variations due to diet.

In the present study, subjects who consumed ≥2 servings of vegetables per day had lower CRP levels, even after adjusting for different covariates. The association between a higher intake of fruit and vegetables and lower CRP levels appears to be consistent in both adults and children [14,15,16,17]. Lee et al. found in 7574 middle-aged Koreans participants in a population-based study that the ‘vegetable’ pattern was inversely associated with CRP [15]. Oliveira described this association only in men [16]. In the Identification and prevention of Dietary- and lifestyle-induced health EFfects In Children and infantS (IDEFICS) study a high-frequency intake of vegetables was inversely related to an inflammatory status in 16,228 children [14]. In a previous study, we found that girls in the highest hs-CRP tertile had a significantly lower intake of vegetables compared to those in tertiles 1 or 2 [17]. The beneficial combination of fiber and antioxidant vitamins, which are contained in vegetables and fruits, may underlie the inverse association with CRP [17].

Fish consumption is a common component of the Mediterranean diet. In our population, the consume ≥3 servings of fish/seafood per week was associated with lower CRP levels. Fish intake is the main dietary source of omega-3 fatty acids. Some studies have associated omega-3 intake with a reduction in plasma biomarkers of inflammation such as CRP [20,21]. Dietary omega-3 were inversely associated with cardiovascular disease incidence [22,23], because in addition to reducing inflammatory markers, they also lower plasma lipid concentration, inhibit plaque formation, decrease platelet aggregation, and reduce arrhythmias risk [24].

The effect of dairy products on inflammatory markers is inconclusive. In fact, it has been reported that dairy products can have a beneficial, neutral, or harmful effect on inflammation [25]. We found that subjects who consume ≥1 serving of butter, margarine, or cream per day had significantly lower CRP levels that those who consume fewer dairy products, even after adjusting by confounders. Similar to our results, the ATTICA study including 3042 healthy Greeks [26] and Salas-Salvado et al. analyzing 772 high cardiovascular risk subjects [13] reported an inverse relationship between the consumption of dairy products and inflammatory markers, including CRP. Finally, in a systematic review of 52 clinical trials investigating inflammatory markers in relation to the consumption of dairy products, Bordoni et al. suggested that dairy products, in particular fermented products, have anti-inflammatory properties in humans not suffering from allergy to milk, in particular in subjects with metabolic disorders [25].

We have not found association between CRP levels and the consumption of nuts or olive oil, two important components of the Mediterranean diet. Although a PREDIMED substudy did not find a relationship between CRP concentration and the consumption of olive oil [13], a recent systematic review concluded that olive oil might exert beneficial effects on CRP, although due to the heterogeneity of the studies a conservative interpretation of the results was recommended [27]. Several studies have investigated the associations between nut consumption and inflammatory biomarkers with inconsistent results. In two large prospective cohorts, nut consumption was inversely associated with CRP concentrations [28]. However, the PREDIMED trial observed a reduction in IL-6 but not CRP in participants consuming a Mediterranean diet with 30 g mixed nuts, compared with the low-fat diet group [29] and a recent meta-analysis suggests that nut consumption have no significant effect on CRP [30].

Our study has several limitations. First, the study was cross-sectional designed and may therefore be susceptible to reverse causality. Second, defining a dietary pattern with a questionnaire including only 14 questions can be inaccurate. Third, the answers of the participants may not be related to their actual intake. Another possible limitation was the lack of information on consumption of antioxidant supplements in our population that could be a potential confounder in the association of the MEDAs with the CRP concentrations. Finally, CRP is only a surrogate marker of inflammation sometimes used as a predictor of cardiovascular risk. Nevertheless, our study has the strength of including a large sample size from a homogeneous population representative of the general population.

Therefore, adherence to a Mediterranean-type diet measured by a simple questionnaire of 14 questions is associated with a decrease in the CRP concentration, even after adjusting by confounders. Some components of this Mediterranean-type diet (higher consumption of vegetables, fruits, dairy products, and fish) seem to be responsible for this association.

## Figures and Tables

**Table 1:** Characteristics of the population by C-reactive protein tertiles.

	All (*n* = 1411)	Tertile 1 (*n* = 471)	Tertile 2 (*n* = 470)	Tertile 3 (*n* = 470)	*p* *
CRP (mg/L); mean ± SD	5.0 ± 6.2	0.9 ± 0.4	3.1 ± 0.8	11.1 ± 7.6	<0.001
Age (years); mean ± SD	61.4 ± 6.0	61.6 ± 5.6	61.5 ± 5.9	61.8 ± 6.1	0.696
Male (%)	42.7	42.3	46.2	39.6	0.407
Alcohol consumption (%)	46.6	43.9	49.5	46.3	0.475
Smoker status (%)					
Current smoker Past smoker Never smoker Hypertension (%)	16.5 36.0 47.4 36.4	15.3 37.4 47.3 32.6	16.2 37.2 46.6 36.2	18.1 33.5 48.4 40.4	0.711 0.012
Hypercholesterolemia (%)	48.0	47.3	51.0	46.1	0.694
Diabetes mellitus (%)	16.7	15.9	16.4	17.9	0.423
Cardiovascular disease (%)	4.6	5.2	4.9	3.7	0.269
Metabolic syndrome (%)	39.8	37.4	37.4	44.5	<0.001
BMI (kg/m^2^); mean ± SD	28.3 ± 4.7	27.9 ± 4.5	28.0 ± 4.4	29.2 ± 4.1	<0.001
Antiplatelet treatment (%)	9.3	9.8	8.7	9.4	0.822
Statin treatment (%)	28.4	30.8	28.9	25.5	0.074
Antidiabetic treatment (%)	9.1	9.4	9.1	8.9	0.821
Hypertension treatment (%)	36.0	33.4	34.9	39.8	0.042

SD: standard deviation. CRP: C-reactive protein. BMI: Body mass index. * *p* trend.

**Table 2:** Multivariable linear regression model to evaluate the association between C-reactive protein concentration and Mediterranean Diet Adherence Screener (MEDAS) score.

Variables	B Regression Coefficient	Confidence Interval 95%	B Standardized Regression Coefficient	*p*
Constant	0.630	−0.162–1.422		0.119
MEDAS score	−0.030	−0.058–(−0.001)	−0.055	0.041
Age	−0.001	−0.012–0.009	−0.007	0.805
Gender (male)	−0.111	−0.234–0.011	−0.048	0.075
Hypertension	0.120	−0.126–0.365	0.050	0.339
Metabolic syndrome	0.061	−0.089–0.211	0.026	0.428
Body mass index	0.027	0.012–0.042	0.112	<0.001
Statins treatment	−0.204	−0.341–(−0.067)	−0.081	0.004
Hypertension treatment	0.025	−0.220–0.270	0.010	0.843

**Table 3:** C-reactive protein concentration depending on the answer to each point of the 14-point Mediterranean Diet Adherence Screener (MEDAS).

	*n* (Yes/No)	CRP (mg/L)		*p*	*p* *
		Yes	No		
1. Do you use olive oil as the principal source of fat for cooking?	41/1364	5.05 (6.17)	5.89 (9.18)	0.657	0.380
2. Do you consume ≥4 tablespoon of olive oil per day?	665/740	4.71 (5.49)	5.37 (6.77)	0.257	0.387
3. Do you consume ≥2 servings of vegetables per day?	625/780	4.96 (6.62)	5.18 (5.98)	0.003	0.003
4. Do you consume ≥3 pieces of fruit per day?	800/605	4.66 (5.64)	5.63 (6.99)	0.004	0.003
5. Do you consume <1 serving of red meat, hamburger, or sausages per day?	1157/248	4.98 (6.23)	5.50 (6.42)	0.311	0.208
6. Do you consume <1 serving of butter, margarine, or cream per day?	1229/176	5.16 (6.28)	4.54 (6.20)	0.018	0.041
7. Do you consume <1 carbonated and/or sugar-sweetened beverages per day?	1201/204	4.99 (6.09)	5.53 (7.24)	0.504	0.396
8. Do you drink ≥7 cups (100 ml) of wine per week?	431/974	4.78 (5.83)	5.22 (6.46)	0.194	0.326
9. Do you consume ≥3 servings of pulses per week?	521/884	5.26 (6.15)	4.97 (6.35)	0.756	0.607
10. Do you consume ≥3 servings of fish/seafood per week?	873/532	4.93 (6.28)	5.33 (6.26)	0.045	0.058
11. Do you consume <2 commercial pastry such as cookies or cake per week?	508/897	5.16 (6.43)	4.94 (5.99)	0.766	0.978
12. Do you consume ≥3 servings of nuts per week?	663/742	4.96 (6.50)	5.19 (6.07)	0.146	0.967
13. Do you prefer to eat chicken, turkey, or rabbit instead of beef, pork, hamburgers, or sausages?	925/480	5.18 (6.55)	4.82 (5.65)	0.728	0.918
14. Do you consume ≥2 times per week boiled vegetables, pasta, rice, or other dishes with a sauce of tomato, garlic, onion, or leeks sautéed in olive oil?	646/759	5.17 (6.36)	4.96 (6.14)	0.814	0.804

Mean (Standard Deviation (SD)). *p* * After adjusting by age, gender, hypertension, metabolic syndrome, body mass index, statin treatment and hypertension treatment.
